# Comprehensive pathogen detection in sera of Kawasaki disease patients by high-throughput sequencing: a retrospective exploratory study

**DOI:** 10.1186/s12887-020-02380-7

**Published:** 2020-10-15

**Authors:** Yuka Torii, Kazuhiro Horiba, Satoshi Hayano, Taichi Kato, Takako Suzuki, Jun-ichi Kawada, Yoshiyuki Takahashi, Seiji Kojima, Yusuke Okuno, Tomoo Ogi, Yoshinori Ito

**Affiliations:** 1 Department of Pediatrics, Nagoya University Graduate School of Medicine, 65 Tsurumai-cho, Showa-ku, Nagoya, 466-8550 Japan; 2 Department of Genetics, Research Institute of Environmental Medicine Nagoya University, Furo-cho, Chikusa-ku, Nagoya, 464-8601 Japan; 3 Department of Human Genetics and Molecular Biology, Nagoya University Graduate School of Medicine, 65 Tsurumai-cho, Showa-ku, Nagoya, 466-8550 Japan; 4 Center for Advanced Medicine and Clinical Research, Nagoya University Hospital, 65 Tsurumai-cho, Showa-ku, Nagoya, 466-8550 Japan

**Keywords:** Kawasaki disease, Pathogen, High-throughput sequencing

## Abstract

**Background:**

Kawasaki disease (KD) is an idiopathic systemic vasculitis that predominantly damages coronary arteries in children. Various pathogens have been investigated as triggers for KD, but no definitive causative pathogen has been determined. As KD is diagnosed by symptoms, several days are needed for diagnosis. Therefore, at the time of diagnosis of KD, the pathogen of the trigger may already be diminished. The aim of this study was to explore comprehensive pathogens in the sera at the acute stage of KD using high-throughput sequencing (HTS).

**Methods:**

Sera of 12 patients at an extremely early stage of KD and 12 controls were investigated. DNA and RNA sequences were read separately using HTS. Sequence data were imported into the home-brew meta-genomic analysis pipeline, PATHDET, to identify the pathogen sequences.

**Results:**

No RNA virus reads were detected in any KD case except for that of equine infectious anemia, which is known as a contaminant of commercial reverse transcriptase. Concerning DNA viruses, human herpesvirus 6B (HHV-6B, two cases) and *Anelloviridae* (eight cases) were detected among KD cases as well as controls. Multiple bacterial reads were obtained from KD and controls. Bacteria of the genera *Acinetobacter*, *Pseudomonas*, *Delfita*, *Roseomonas*, and *Rhodocyclaceae* appeared to be more common in KD sera than in the controls.

**Conclusion:**

No single pathogen was identified in serum samples of patients at the acute phase of KD. With multiple bacteria detected in the serum samples, it is difficult to exclude the possibility of contamination; however, it is possible that these bacteria might stimulate the immune system and induce KD.

## Background

Kawasaki disease (KD) is an idiopathic systemic vasculitis that predominantly damages coronary arteries in children. Coronary artery lesions, such as coronary artery dilation or coronary aneurysms, are severe sequelae that may result in stenosis or obstruction. In severe cases, fatal outcomes may result from ischemic heart disease or coronary aneurysm rupture. Epidemiological studies suggest that both genetic and environmental factors, including pathogens, are involved in the pathophysiology of KD [1, 2]. Regarding genetic factors, genome-wide association studies have identified susceptibility loci for KD [3, 4]. Meanwhile, environmental factors have been explored from various points of view and several pathogens have been proposed as triggers. *Streptococci*, *Staphylococci*, *Chlamydia*, *Mycoplasma*, adenovirus, Epstein-Barr virus, and parvovirus B19 have all been reported as candidate pathogens [1]. Furthermore, several epidemiological studies report the association between KD and epidemics of illness [5, 6]. These studies suggest that KD is caused or elicited by infection. However, no pathogen has been definitively determined as the causative pathogen of KD; in other words, the etiologic agent and immunopathogenesis of KD remain unknown. Of note, there are etiological substances that induce inflammation in each KD clinical manifestation, such as coronary artery lesions [7]. Histopathological findings and studies on animal models have suggested that immune responses to certain substances, such as superantigens, heat shock protein 60, RNA viruses, and pathogen-associated molecular patterns, may be involved in the onset of KD [1]. These data suggest that inflammation may be induced by various infectious agents. Investigating numerous pathogens using clinical specimens is challenging because sample availability is usually limited. Moreover, blood samples from patients at the acute stage are rarely examined, as a confirmed diagnosis of KD requires several days.

High-throughput sequencing (HTS) has recently been applied in metagenomic approaches for various diseases. Direct identification of genome sequences enables non-targeted, comprehensive pathogen detection in clinical specimens. We previously analyzed sera from patients with encephalitis, acute liver failure/fulminant hepatitis, bloodstream infection, or myocarditis with unknown etiology, and identified the viral and bacterial genomes of presumptive pathogens of these diseases [8–10]. In the present study, sera of patients at an extremely early stage of KD were investigated to detect pathogen genomes using HTS.

## Methods

### Patients and samples

Twelve KD patients were included in this study. Written informed consent was provided by patients and/or their guardians. Blood samples were collected simultaneously at their first visit to the clinic as a part of routine laboratory investigations. As KD is a syndrome, patients suspected to have the disease were hospitalized and an investigation performed to exclude other diseases. These patients were observed until the clinical criteria for KD were met. This study was designed with the intention of obtaining patient blood samples prior to KD diagnosis. Sera were separated and stored at − 80 °C until use. Sera from 12 control individuals were also investigated. The control group comprised of two subgroups; five healthy adults, two females and three males of median age 34 years (HC; healthy control), and seven afebrile children, three females and four males of median age 8 years, with chronic disease (AC; afebrile control). All study protocols were approved by the Institutional Review Board of Nagoya University Graduate School of Medicine (permission number: 2014–0261).

For KD diagnosis, clinicians followed the guidelines set by the Ministry of Health, Labour, and Welfare of Japan [11]. The clinical criteria for KD included six major clinical findings: 1. Persistent fever for 5 days or more (inclusive of cases in which fever subsided before the fifth day in response to therapy); 2. Bilateral conjunctival congestion; 3. Changes in the lips and oral cavity (reddening of lips, strawberry tongue, diffuse injection of oral and pharyngeal mucosa); 4. Polymorphous exanthema; 5. Changes in peripheral extremities (initial stage: reddening of palms and soles, indurative edema; convalescent stage: membranous desquamation from fingertips); and 6. Acute non-purulent cervical lymphadenopathy. Patients were diagnosed with KD if they fulfilled five or more of the six clinical criteria. Coronary artery lesions were investigated in all patients by echocardiography when clinicians diagnosed or suspected KD prior to treatment initiation.

### High-throughput sequencing

RNA and DNA from the serum of each patient were sequenced. RNA was extracted from 200 μL of serum using the NucleoSpin Blood Kit (Macherey-Nagel, Düren, Germany). Thereafter, cDNA was synthesized and amplified as previously described [9]. DNA was extracted from 400 μL of serum using the QIAamp UCP Pathogen Mini Kit (Qiagen, Hilden, Germany). To prepare sequencing libraries, 1 ng of DNA or cDNA was used with the Nextera XT DNA Sample Preparation Kit (Illumina, San Diego, CA) [8–10]. The library quality was analyzed using Bioanalyzer (Agilent Technologies, Santa Clara, CA) and quantified using the QX200 Droplet Digital PCR System (Bio-Rad, Hercules, CA). DNA and RNA libraries were sequenced on the HiSeq 2500 System (Illumina) using the 2× 150-bp paired-end sequencing protocol in the rapid run mode.

### Data analysis

To identify pathogen-derived sequences, sequence data were imported into the meta-genomic analysis pipeline PATHDET [12]. PATHDET was used for taxonomic classification from sequences not mapped to the human reference genome following the removal of adapter sequences and low-quality sequences. Pathogen-derived sequences were reported based on previously established threshold criteria [8]. Results from PATHDET are shown according to taxonomic hierarchy; family, genus, and species. The abundance is represented by reads per million reads (RPM), the number of reads per million sequencing reads; and relative abundance (RA), the relative abundance of microorganisms. For RNA library data, de novo assembly was performed using the cloud-computing pipeline VirusTAP (National Institute of Infectious Diseases, Japan) [13] in addition to PATHDET. Contigs not mapped to the RefSeq genome database were compared between one sample and other samples to explore suspected novel pathogen sequences using CLC Genomics Workbench 12.0 (CLC Bio; Qiagen).

## Results

Serum samples were obtained on day 2 of fever onset in most patients (Table 1). The clinical criteria for KD were met in all patients.

**Table 1:** Patient characteristics

Patient ID	Age Range (year)	Days of fever onset at sampling^c^	KD symptoms ^a^ at sampling	Sequela
KD1	0–1	1	14	–
KD2	1–5	2	1345	–
KD3	5–10	2	16	NA
KD4	0–1	4	123,456	–
KD5	1–5	3	123,456	–
KD6	5–10	2	16	CAL transient^b^
KD7	5–10	3	16	–
KD8	1–5	2	126	–
KD9	1–5	2	12,345	CAL transient^b^
KD10	0–1	2	1	–
KD11	1–5	2	12,346	–
KD12	0–1	2	16	–

^a^ KD symptoms: 1 = Fever, 2 = Conjunctivitis, 3 = Mucositis, 4 = Rash, 5 = Extremity changes, 6 = Lymphadenopathy; clinical criteria for KD were met in all patients

^b^Coronary artery lesions subsided within a year

^c^ The day of fever onset was considered day 1

NA (not available), *CAL* Coronary artery lesion.

None of the patients received antibiotics before blood samples were collected. For RNA sequences, an average of 11,567,957 reads per sample were obtained from KD patients. No RNA virus reads were detected in any KD case except for equine infectious anemia virus, which is known to be a contaminant of commercial reverse transcriptase [14]. Assuming that a specific and unknown pathogen induces KD, we searched for common sequences between contigs that were not mapped to the RefSeq genome database. However, there was no common sequence between KD patients and controls.

For DNA sequences, the average of available reads per sample were 28,919,236 (KD patients), 20,804,952 (HC), and 13,354,215 (AC), respectively. Regarding the DNA virus analysis, human herpesvirus 6B (HHV-6B) was detected in two of the 12 KD patients and one of the seven AC children, respectively. *Anelloviridae* was detected in eight out of 12 KD patients and four out of seven AC children (Additional File 1). In the bacterial analysis, numerous bacterial reads were detected in both KD patients and controls, suggesting bacterial contamination of samples during the experimental process. Therefore, candidate bacterial pathogens were defined as those with more than 100 RPM available at the genus level and RA greater than 0.1 at the genus or species levels. Pathogens of the genera *Acinetobacter*, *Pseudomonas*, *Delfita*, *Roseomonas,* and *Rhodocyclaceae* satisfied this definition (Table 2). Among these genera, *Acinetobacter soli*, *Pseudomonas kribensis*, and *Rhodocyclaceae bacterium* Paddy-1 could be identified at the species level.

**Table 2:** Number of DNA sequence reads and susceptible bacteria

ID	Available read	Human reads (RPM)	Pathogen reads (RPM)	Susceptible pathogens (RPM, RA)
KD1	38,321,575	998,404	578	*Delftia* (173.5, 0.30)***Rhodocyclaeceae bacterium*** **Paddy-1 (104.9, 0.18)**
KD2	37,562,658	998,930	365	***Acinetobacter soli*** **(143.7, 0.39)**
KD3	39,025,894	999,530	110	NA
KD4	28,919,236	998,950	313	NA
KD5	67,166,028	997,211	1867	***Delftia acidovorans*** **(242.0, 0.13)** ***Pseudomonas kribbensis*** **(199.8, 0.11)**
KD6	26,744,455	999,051	239	NA
KD7	20,929,225	997,964	663	*Pseudomonas* (110.1, 0.17)***Roseomonas*** **sp. FDAARGOS_362 (90.0, 0.14)**
KD8	30,025,914	999,318	221	***Acinetobacter soli*** **(90.3, 0.41)**
KD9	22,413,817	999,062	385	***Acinetobacter soli*** **(170.4, 0.44)**
KD10	17,319,029	996,787	2047	***Acinetobacter soli*** **(832.2, 0.41)** ***Pseudomonas kribbensis*** **(194.8, 0.10)**
KD11	18,716,804	999,331	195	NA
KD12	22,309,536	999,609	57	NA
HC1	20,804,952	989,280	181	NA
HC2	15,888,618	963,349	621	NA
HC3	21,192,125	999,500	45	NA
HC4	24,983,027	999,016	190	*Pseudomonas* (122.0, 0.64)
HC5	15,820,231	997,747	228	NA
AC1	25,702,148	998,293	428	NA
AC2	12,464,308	995,548	1110	NA
AC3	2,723,604	997,081	350	NA
AC4	19,385,648	995,158	812	*Staphylococcus* (101.2, 0.13)
AC5	10,409,172	998,455	257	NA
AC6	13,844,182	999,064	364	***Delftia acidovorans*** **(54.8, 0.15)**
AC7	8,950,442	998,940	199	NA

A susceptible pathogen is defined as follows: Pathogen genus group of RPM > 100 and pathogen rank group of RA > 0.1 and pathogen rank group is listed as genus or species (bold) level.

*RPM* Read per Million, *RA* Relative Abundance.

## Discussion

Since the first report of KD in 1967, multiple hypotheses have been made on the cause of the disease. As KD is a syndrome, its diagnosis is usually not confirmed until the fever has lasted for 5 days and other clinical findings meet the criteria. Other symptoms of KD, such as rash and cervical lymphadenopathy, often appear a few days following fever onset. By this time, the immune system is thought to have already eliminated the trigger pathogen. This is one of several speculations as to why pathogens have not been detected using current diagnostic methods. Therefore, in this study, we attempted to investigate blood samples at an extremely early stage of the disease. The median day of blood sampling was day 2 from fever onset. However, even though we studied the early stage of the disease, no common pathogen could be identified as the cause of KD using HTS.

HHV-6B was detected in two patients with KD and one AC child. HHV-6B is a ubiquitous virus and primary HHV-6B infection frequently occurs between 6 months and 3 years of age [15]. KD is also common in these age groups. It is unlikely that HHV-6B induced KD because most children with HHV-6B infection did not present with KD symptoms. Additionally, HHV-6 DNA is known to persist in most children intermittently following primary infection [16]. As for the patients KD2 and KD6, our interpretation is that they did not have HHV-6 primary disease but had typical KD, because they fulfilled the required criteria for KD. Moreover, KD2 and KD6 were over 3 years old, which is relatively older compared to most children with HHV-6B infection. Otherwise, the immune response for HHV-6B may be affected in KD patients [17, 18]. *Anelloviridae* were found in eight patients with KD and four AC children in the present study. *Anelloviridae* has not been fully recognized because of its difficulty in culture. It has become well recognized by HTS and is now known to occupy a large fraction of the human serum virome [19, 20]. In a previous report, torque teno virus 7, which belongs to the *Anelloviridae* family, was detected in KD patients, but not in controls [21]. However, no specific species of the *Anelloviridae* family for KD were found in this study. Moreover, the levels of *Anelloviridae* reads detected in two AC children were higher than that in others, including KD patients. Here, multiple bacteria were detected in sera of KD patients but were also found in sera of healthy controls, suggesting the possibility of sample contamination. DNA contamination might be ubiquitous in nucleic acid extraction and/or library preparation [22], and thus more challenging to work with samples of low microbial biomass, e.g., blood, in comparison to samples of high microbial biomass e.g., feces. To correct for the influence of contaminating bacterial DNA, bacterial characteristics were compared between KD patients, and controls consisting of subgroups. Unfortunately, no bacteria were detected exclusively in the KD patients and not in the controls. However, bacteria of the genera *Acinetobacter*, *Pseudomonas*, *Delfita*, *Roseomonas*, and *Rhodocyclaceae* were more common in KD samples than in control samples. These bacteria are water- and soil-associated bacteria and representative of the contaminating sequence; however, *Acinetobacter*, *Pseudomonas*, *Delfita*, and *Roseomonas,* which exist as normal flora in the skin, oral tract, or intestine, might be able to induce bloodstream infections in immunocompromised patients [23–26]. They could also possibly enter the bloodstream and stimulate the immune system of immunocompetent children and trigger KD. It is not surprising that some bacterial strains in the microbiota can be detected in the KD and control groups regardless of the samples’ contamination status and of RPM. We hypothesize that although some bacteria present in the normal flora (in the microbiota) can easily invade the host, inflammation should be rare since not only these strains have a low-virulent nature, but there may also be tolerance mechanisms in place [27, 28]. Of note, Rhim et al. [29] suggested that KD may be associated with bacterial species in the normal flora that may be influenced by environmental changes. Additionally, we comprehensively examined RNA viruses in the present study. Although our HTS method previously revealed the profile of an RNA virus in serum samples from patients with acute liver failure/fulminant hepatitis and myocarditis [9, 10], no pathogenic RNA virus could be detected from sera in the present study. Recent studies support the hypothesis that KD pathogenesis is closely associated with dysregulation of immune responses to various viruses or microbes [30, 31]. More studies are needed to identify specific associations between detected pathogens and their ability to act as a trigger for immune system activation in KD cases.

There has been an increasing number of reports regarding the associations between severe acute respiratory syndrome coronavirus 2 (SARS-CoV-2) and KD-like symptoms; e.g. the multisystem inflammatory syndrome in children (MIS-C) [32]. It seems to be different from typical KD, in that it presents with cardiogenic shock; however, there are some undeniable common points between two diseases in symptoms in a recent publication [33]. A previous study reported the association of coronavirus other than SARS-CoV-2 and KD prior to the Covid-19 pandemic [34]. In our study, no reads for coronaviruses, including that of SARS-CoV-2, were detected. Although it is known that SARS-CoV-2 can be detected in sera [35], serum was exclusively investigated and no samples derived from the upper respiratory tract were studied here. It is proposed that the etiology of KD as one of postinfectious immune-mediated diseases may be related to certain strains within the human microbiota; moreover, KD and other infection-related immune-mediated diseases such as acute rheumatic fever may be elicited by substances derived from infected cells, including toxins, pathogen-associated molecular patterns (PAMPs), damage-associated molecular patterns (DAMPS), and pathogenic proteins and peptides [29]. MIS-C may also have a similar etiology. Of note, MIS-C cases have been reported in Western countries, but not in Eastern Asian countries such as Japan and Korea where KD is more endemic [36, 37]. It is known that the microbiota is different in distinct ethnic groups; moreover, environmental factors such as the diet and antibiotic therapy, also impact the microbiota composition. Thus, it is possible that the impacts of COVID-19 such as the shutdown of schools, and the consequent diet change can affect the transient dysbiosis in MIS-C [37].

Several studies have previouslyreported data on pathogen detection for KD using HTS. L’Huillier et al. [38] described multiple viruses that were detected using HTS in seven confirmed children with KD. Their study had an advantage over our study for the detection causative viruses because the number of sequence reads in their study was much higher compared to ours. The data on the variety of TTV detected in KD patients were similar to our study. Moreover, their study described vaccine-origin viruses in two patients. Hamada et al. [39] described a case with four recurrent KD episodes that may be associated with *Streptococcus* spp. Thissen et al. [21] described the association of TTV-7 in KD patients. However, none of these studies have reported the specific pathogens for KD.

There are several limitations to this study. First, the sample sizes of the patients and controls are somewhat small to robustly confirm the significance of the results. Thus, further studies are needed with other methods, such as 16 s rRNA sequencing. Second, the controls are heterogeneous compared with the patient group. Moreover, serum samples were exclusively analyzed expecting specific viruses that cause viremia in patients with KD. However, the local viral infection could be the trigger for KD. Respiratory viruses mainly infect the respiratory tract and are usually not detected in blood samples.

## Conclusions

In conclusion, using HTS, no specific single pathogen was identified in blood samples of patients at the acute stage of KD. Multiple bacteria were detected in the blood of KD patients; although it is difficult to exclude the possibility of contamination, it is possible that these bacteria might stimulate the immune system and induce KD.

## Supplementary information

**Additional file 1.** Virus detection in DNA sequence. The data shows virus reads detected in each sample. The value is normalized to the number of aligned reads per million available reads (RPM).

## Data Availability

Nucleotide sequence data reported are available in the DDBJ/EMBL/GenBank databases under the accession number DRA010888.

## Abbreviations

KD: Kawasaki disease
HTS: High-throughput sequencing
HHV-6B: Human herpesvirus 6B
RPM: Reads per million
RA: Relative abundance
SARS-CoV-2: Severe acute respiratory syndrome coronavirus 2

## References

[1] Nagata S (2019). Causes of Kawasaki disease-from past to present. Front Pediatr.

[2] Rowley AH (2018). Is Kawasaki disease an infectious disorder?. Int J Rheum Dis.

[3] Lee YC, Kuo HC, Chang JS, Chang LY, Huang LM, Chen MR (2012). Two new susceptibility loci for Kawasaki disease identified through genome-wide association analysis. Nat Genet.

[4] Onouchi Y, Ozaki K, Burns JC, Shimizu C, Terai M, Hamada H (2012). A genome-wide association study identifies three new risk loci for Kawasaki disease. Nat Genet.

[5] Burns JC, Cayan DR, Tong G, Bainto EV, Turner CL, Shike H (2005). Seasonality and temporal clustering of Kawasaki syndrome. Epidemiology.

[6] Yanagawa H, Nakamura Y, Yashiro M, Fujita Y, Nagai M, Kawasaki T (1988). A nationwide incidence survey of Kawasaki disease in 1985-1986 in Japan. J Infect Dis.

[7] Lee KY, Rhim JW, Kang JH (2012). Kawasaki disease: laboratory findings and an immunopathogenesis on the premise of a "protein homeostasis system". Yonsei Med J.

[8] Horiba K, Kawada JI, Okuno Y, Tetsuka N, Suzuki T, Ando S (2018). Comprehensive detection of pathogens in immunocompromised children with bloodstream infections by next-generation sequencing. Sci Rep.

[9] Suzuki T, Kawada JI, Okuno Y, Hayano S, Horiba K, Torii Y (2017). Comprehensive detection of viruses in pediatric patients with acute liver failure using next-generation sequencing. J Clin Virol.

[10] Takeuchi S, Kawada JI, Okuno Y, Horiba K, Suzuki T, Torii Y (2018). Identification of potential pathogenic viruses in patients with acute myocarditis using next-generation sequencing. J Med Virol.

[11] Ayusawa M, Sonobe T, Uemura S, Ogawa S, Nakamura Y, Kiyosawa N (2005). Revision of diagnostic guidelines for Kawasaki disease (the 5th revised edition). Pediatr Int.

[12] Horiba K (2019). PATHDET: A pathogen detection tool through sequence data from in and ex vivo enviroments. PATHDET v1.0.

[13] Yamashita A, Sekizuka T, Kuroda M (2016). VirusTAP: viral genome-targeted assembly pipeline. Front Microbiol.

[14] Wally N, Schneider M, Thannesberger J, Kastner MT, Bakonyi T, Indik S (2019). Plasmid DNA contaminant in molecular reagents. Sci Rep.

[15] Zerr DM, Meier AS, Selke SS, Frenkel LM, Huang ML, Wald A (2005). A population-based study of primary human herpesvirus 6 infection. N Engl J Med.

[16] Caserta MT, McDermott MP, Dewhurst S, Schnabel K, Carnahan JA, Gilbert L (2004). Human herpesvirus 6 (HHV6) DNA persistence and reactivation in healthy children. J Pediatr.

[17] Kawano Y, Kawada JI, Nagai N, Ito Y (2019). Reactivation of human herpesviruses 6 and 7 in Kawasaki disease. Mod Rheumatol.

[18] Okano M, Luka J, Thiele GM, Sakiyama Y, Matsumoto S, Purtilo DT (1989). Human herpesvirus 6 infection and Kawasaki disease. J Clin Microbiol.

[19] De Vlaminck I, Khush KK, Strehl C, Kohli B, Luikart H, Neff NF (2013). Temporal response of the human virome to immunosuppression and antiviral therapy. Cell.

[20] Focosi D, Antonelli G, Pistello M, Maggi F (2016). Torquetenovirus: the human virome from bench to bedside. Clin Microbiol Infect.

[21] Thissen JB, Isshiki M, Jaing C, Nagao Y, Lebron Aldea D, Allen JE (2018). A novel variant of torque Teno virus 7 identified in patients with Kawasaki disease. PLoS One.

[22] Salter SJ, Cox MJ, Turek EM, Calus ST, Cookson WO, Moffatt MF (2014). Reagent and laboratory contamination can critically impact sequence-based microbiome analyses. BMC Biol.

[23] Bilgin H, Sarmis A, Tigen E, Soyletir G, Mulazimoglu L (2015). Delftia acidovorans: a rare pathogen in immunocompetent and immunocompromised patients. Can J Infect Dis Med Microbiol.

[24] Kimura K, Hagiya H, Nishi I, Yoshida H, Tomono K (2018). Roseomonas mucosa bacteremia in a neutropenic child: a case report and literature review. IDCases.

[25] Potron A, Poirel L, Nordmann P (2015). Emerging broad-spectrum resistance in Pseudomonas aeruginosa and Acinetobacter baumannii: mechanisms and epidemiology. Int J Antimicrob Agents.

[26] Diekema DJ, Hsueh PR, Mendes RE, Pfaller MA, Rolston KV, Sader HS, et al. The Microbiology of Bloodstream Infection: 20-Year Trends from the SENTRY Antimicrobial Surveillance Program. Antimicrob Agents Chemother. 2019;63(7):e00355–19. 10.1128/AAC.00355-19 PMC6591610 31010862

[27] Vaishnavi C (2013). Translocation of gut flora and its role in sepsis. Indian J Med Microbiol.

[28] Castillo DJ, Rifkin RF, Cowan DA, Potgieter M (2019). The healthy human blood microbiome: Fact or fiction?. Front Cell Infect Microbiol.

[29] Rhim JW, Kang HM, Han JW, Lee KY (2019). A presumed etiology of Kawasaki disease based on epidemiological comparison with infectious or immune-mediated diseases. Front Pediatr.

[30] Hara T, Nakashima Y, Sakai Y, Nishio H, Motomura Y, Yamasaki S (2016). Kawasaki disease: a matter of innate immunity. Clin Exp Immunol.

[31] Nakamura A, Ikeda K, Hamaoka K (2019). Aetiological significance of infectious stimuli in Kawasaki disease. Front Pediatr.

[32] Riphagen S, Gomez X, Gonzalez-Martinez C, Wilkinson N, Theocharis P (2020). Hyperinflammatory shock in children during COVID-19 pandemic. Lancet.

[33] Licciardi F, Pruccoli G, Denina M, Parodi E, Taglietto M, Rosati S (2020). SARS-CoV-2-induced Kawasaki-like Hyperinflammatory syndrome: a novel COVID phenotype in children. Pediatrics.

[34] Shirato K, Imada Y, Kawase M, Nakagaki K, Matsuyama S, Taguchi F (2014). Possible involvement of infection with human coronavirus 229E, but not NL63, in Kawasaki disease. J Med Virol.

[35] Zheng S, Fan J, Yu F, Feng B, Lou B, Zou Q (2020). Viral load dynamics and disease severity in patients infected with SARS-CoV-2 in Zhejiang province, China, January-march 2020: retrospective cohort study. Bmj.

[36] Kim Y-J, Park H, Choi YY, Kim YK, Yoon Y, Kim K-R (2020). Defining association between covid-19 and the multisystem inflammatory syndrome in children through the pandemic. J Korean Med Sci.

[37] Lee K-Y, Rhim J-W, Kang J-H (2020). Immunopathogenesis of COVID-19 and early immunomodulators. Clin Exp Pediatr.

[38] L'Huillier AG, Brito F, Wagner N, Cordey S, Zdobnov E, Posfay-Barbe KM (2019). Identification of viral signatures using high-throughput sequencing on blood of patients with Kawasaki disease. Front Pediatr.

[39] Hamada H, Sekizuka T, Oba K, Katano H, Kinumaki A, Terai M (2016). Comprehensive pathogen detection associated with four recurrent episodes of Kawasaki disease in a patient during a single year using next-generation sequencing. JMM Case Rep.

